# Anaphylaxis cases presenting to primary care paramedics in Quebec

**DOI:** 10.1002/iid3.78

**Published:** 2015-08-16

**Authors:** Nofar Kimchi, Ann Clarke, Jocelyn Moisan, Colette Lachaine, Sebastien La Vieille, Yuka Asai, Lawrence Joseph, Chris Mill, Moshe Ben‐Shoshan

**Affiliations:** ^1^ Technion American Medical Students ProgramHaifaIsrael; ^2^ Division of Rheumatology, Department of MedicineUniversity of CalgaryCalgary, AlbertaCanada; ^3^ Directeur Médical Régional des Services Préhospitaliers D'urgence de L'OutaouaisQuebecCanada; ^4^ Directrice médicale nationaleDirection Adjointe de Services Préhospitaliers D'urgenceMSSS, QuebecCanada; ^5^ Food DirectorateHealth CanadaOttawa, OntarioCanada; ^6^ Division of Dermatology, Department of MedicineQueen's UniversityKingston, OntarioCanada; ^7^ Department of Epidemiology and BiostatisticsMcGill UniversityMontreal, QuebecCanada; ^8^ School of Population and Public HealthUniversity of British ColumbiaVancouver, British ColumbiaCanada; ^9^ Division of Allergy and Clinical Immunology, Department of MedicineMcGill University Health CentreMontreal, QuebecCanada

**Keywords:** Anaphylaxis, Emergency Medical Services, epinephrine, management, triggers

## Abstract

Data on anaphylaxis cases in pre‐hospital settings is limited. As part of the Cross Canada Anaphylaxis Registry (C‐CARE), we assessed anaphylaxis cases managed by paramedics in Outaouais, Quebec. A software program was developed to prospectively record demographic and clinical characteristics as well as management of cases meeting the definition of the anaphylaxis. Univariate and multivariate logistic regressions were compared to assess factors associated with severity of reactions and epinephrine use. Among 33,788 ambulance calls of which 23,486 required transport, 104 anaphylaxis cases were identified (anaphylaxis rate of 0.31% [95%CI, 0.25%, 0.37%] among all ambulance calls and 0.44% [95%CI, 0.36%, 0.54%] among those requiring transport). The median age was 46.8 years and 41.3% were males. The common triggers included food (32.7% [95%CI, 24.0%, 42.7%]), drugs (24.0% [16.4%, 33.6%]), and venom (17.3% [10.8%, 26.2%]). Among all reactions, 37.5% (95%CI, 28.4%, 47.6%) were severe. Epinephrine was not administered in 35.6% (95%CI, 26.6%, 45.6%) of all cases. Males were more likely to have severe reactions (Odds ratio [OR]: 2.50 [95%CI, 1.03, 6.01]). Venom‐induced reactions and severe anaphylaxis were more likely to be managed with epinephrine (OR: 6.9 [95%CI, 1.3, 35.3] and 4.2 [95%CI, 1.5, 12.0], respectively). This is the first prospective study evaluating anaphylaxis managed by paramedics. Anaphylaxis accounts for a substantial proportion of the cases managed by paramedics in Outaouais, Quebec and exceeds prior reports of the proportion of Quebec emergency room visits attributed to anaphylaxis. Although guidelines recommend prompt use of epinephrine for all cases of anaphylaxis, more than a third of cases did not receive epinephrine. It is crucial to develop educational programs targeting paramedics to promote the use of epinephrine in all cases of anaphylaxis regardless of the specific trigger.

## Introduction

While anaphylaxis is a growing societal and individual burden, many knowledge gaps exist regarding the rates, triggers, and management in different settings 1, 2, 3, 4. To date, data on anaphylaxis are usually collected retrospectively, which may lead to information bias 5, 6. Moreover, very few studies have assessed anaphylaxis treatments in the pre‐hospital setting by paramedics (referred hereafter as the Emergency Medical Services or EMS) and none of these studies collected data prospectively 5, 6. Further, no study evaluated the rates, triggers, and management of anaphylaxis in emergency department (ED) versus EMS settings using comparable and rigorous data collection strategies.

The Cross‐Canada Anaphylaxis Registry (C‐CARE) is a novel cross‐country initiative, which aims to bridge knowledge gaps related to the rates, triggers, and management of anaphylaxis. We recently reported on anaphylaxis in pediatric and adult EDs in Quebec 7, 8. Our results revealed that almost 0.3% of all pediatric and adult ED visits were due to anaphylaxis and that the majority of the cases were food induced 7, 8. We also noted underuse of epinephrine, especially in mild and moderate cases, and in adults 7, 8. To broaden our understanding of anaphylaxis in other settings, we extended C‐CARE to evaluate cases of anaphylaxis presenting to the EMS in the Outaouais region of Quebec, Canada.

## Methods

A software program was developed to record information on all suspected allergic reactions. All EMS providers in the Outaouais region were trained by our research team to use portable tablet computers incorporating the program to record any case of suspected allergic reaction or use of epinephrine. The treating paramedic provided information on age, sex, clinical background (presence of co‐morbidities including cardiovascular disease and atopy, medication use, exercise within the 2 h preceding the reaction), clinical characteristics of the reaction (suspected trigger, symptoms, route of exposure, time interval between exposure and development of clinical symptoms), and management (use of epinephrine, antihistamines, steroids, and other medications and the need for admission).

These questionnaires were then evaluated by our team to establish if the patient met the criteria for anaphylaxis 9. In addition, a trained member of our research team in the Outaouais reviewed all cases daily to ensure that no cases of anaphylaxis were missed. Data for missed cases was retrieved through review of the EMS records. Anaphylaxis severity was classified according to a modified grading system published by Brown 10. Mild anaphylaxis was defined when patients presented with skin and subcutaneous tissues symptoms (urticaria, erythema, and angioedema) as well as oral pruritus, nausea (i.e., gastrointestinal involvement) or nasal congestion, sneezing, rhinorrhea, throat tightness (i.e., respiratory involvement). Moderate anaphylaxis was characterized by the presence of any of the previous symptoms as well as crampy abdominal pain, diarrhea, or recurrent vomiting, dyspnea, stridor, cough, wheeze, or “light headedness.” Severe anaphylaxis were defined if symptoms included cyanosis, hypoxia (saturation<92%), respiratory arrest, hypotension, dysrhythmia, confusion, or loss of consciousness 10. Univariate and multivariate logistic regression models were fit to estimate the associations between patient's demographics and clinical characteristics and (1) use of epinephrine and (2) reaction severity. All parameters were estimated using R version 2.12.0 (2010‐10‐15).

The study was approved by the McGill University Health Center Ethic Review Board.

## Results

Among 33,788 ambulance calls between 10 May 2013 and 10 May 2014, 23,486 required transport. There was a total of 104 anaphylaxis cases, which corresponds to 0.31% (95%CI, 0.25%, 0.37%) of all calls and 0.44% (95%CI, 0.37%, 0.54%) of those requiring transport. Almost 90% of data were collected prospectively. The median age was 46.8 years and the majority were females (Table 1). More than a third of cases were severe. Food, mainly peanut, was the main trigger, followed by drugs and venom. Almost one fifth of anaphylaxis cases were related to an unknown cause (Table 2). Most of the reactions were not associated with exercise and almost half occurred at home (Table 1).

**Table 1:** Demographics and co‐morbidities of patients

Variable	% (95%CI)
Number of ambulance calls	33,788
Number of ambulance calls requiring transport	23,486
Number of patients with anaphylaxis	104
% Anaphylaxis among all calls	0.31 (0.25, 0.37)
% Anaphylaxis among calls requiring transport	0.44 (0.37, 0.54)
Median age, years	46.8 (21.3, 60.6)
Adults (> = 18 years)	78.8 (69.5, 86.0)
Males	41.3 (31.9, 51.4)
At home	57.7 (47.6, 67.2)
Not associated with exercise	66.3 (56.3, 75.1)
Severity	
Severe	37.5 (28.4, 47.6)
Moderate	50.0 (40.6, 59.4)
Known asthma	16.3 (10.1, 25.2)
Known food allergy	17.3 (10.8, 26.2)
Known ischemic heart disease	3.8 (1.2, 10.1)
Use of beta‐blockers	4.81 (8, 11.4)
Use of anti‐depressants	5.8 (2.4, 12.6)
Use of angiotensin converting enzyme inhibitors	1.9 (0.3, 7.5)
Use of non‐steroidal anti‐inflammatory	8.7 (4.3, 16.2)

**Table 2:** Reaction triggers

Trigger	% (95%CI)
Food trigger	32.7 (24.0, 42.7)
Peanuts^1^	23.5 (11.4, 41.6)
Tree nuts	2.9 (0.2, 17.1)
Nuts (Not clear if peanuts or tree nuts)	20.6 (9.3, 38.4)
Milk	5.9 (1.0, 21.1)
Shellfish	8.8 (2.3, 24.8)
Fish	17.6 (7.4, 35.2)
Sesame	2.9 (0.2, 17.1)
Multiple food allergens	2.9 (0.2, 17.1)
Unknown food allergens	5.9 (1.0, 21.1)
Other food allergens	20.6 (9.3, 38.4)
Venom	17.3 (10.8, 26.2)
Drugs	24.0 (16.4, 33.6)
Unknown	18.3 (11.6, 27.3)
Other	7.7 (3.6, 15.0)

1 Among all 34 food triggered reactions.

Epinephrine was administered either before or after ambulance arrival in 64.4% of patients (Table 3). In 10.6% of the patients, epinephrine was administered both before and after ambulance arrival (Table 3). Epinephrine was not administered either before or after ambulance arrival in 28.6% of moderate or severe cases. Further, among those with a known food allergy, only 50% used their epinephrine auto‐injector prior to ambulance arrival. Antihistamines and steroids were taken prior to ambulance arrival in 40% and in 3.8% of the cases, respectively, and were not administered after by paramedics (Table 3). There were no fatalities among the 104 cases of anaphylaxis.

**Table 3:** Use of epinephrine, antihistamines, and steroids in the management of anaphylaxis

Variable	% (95%CI)
% of patients with anaphylaxis administered epinephrine prior to OR after EMS arrival	64.4 (54.4, 73.4)
% of patients with anaphylaxis administered epinephrine prior to AND after EMS arrival	10.6 (5,7, 18.5)
% of patients with anaphylaxis not administered epinephrine	35.6 (26.6, 45.6)
% of patients with severe/moderate reactions not administered epinephrine	28.6 (19,8, 39.1)
% of patients with anaphylaxis receiving antihistamines prior to EMS arrival	40.0 (31.0, 50.5)
% of patients with anaphylaxis receiving antihistamines after EMS arrival	0
% of patients with severe/moderate reactions treated with antihistamines	39.6 (29.6, 50.3)
% of patients with anaphylaxis receiving steroids prior to EMS arrival	3.8 (1.2, 10.1)
% of patients with anaphylaxis receiving steroids after EMS arrival	0
% of patients with severe/moderate reactions treated with steroids	3.3 (0.9, 10.0)

Epinephrine use was associated with venom‐induced anaphylaxis and severe reactions (adjusted OR 6.9 [95%CI, 1.3, 35.3] and 4.2 [95%CI, 1.5, 12.0], respectively). The only factor associated with severe reactions was male gender (adjusted OR 2.50 [95%CI, 1.03, 6.01]).

## Discussion

This is the first prospective study assessing anaphylaxis in EMS settings. Our study reveals a high percentage of anaphylaxis cases presenting to EMS in Quebec. In addition, our results reveal a relatively high percentage of venom‐induced anaphylaxis in the EMS and underuse of epinephrine especially in mild anaphylaxis.

The percentage of anaphylaxis among all EMS calls in our study were higher than percentages reported by our group in a tertiary care pediatric center (difference: 0.1% [95%CI, 0. 03%, 0.2%] and comparable to adult centers [0.05% [−0.03%, 0.1%]). However, among all actual transports, the percentage of anaphylaxis was higher in the EMS compared to both pediatric and adult centers (difference: 0.23% [95%CI, 0.14%, 0.33%] and 0.18% [95%CI, 0.08%, 0.28%], respectively) but comparable to a previous study assessing anaphylaxis cases treated by paramedics (−0.000006% [−0.0009%, 0.0009%]) (Table 4) 6. This is not surprising given the differences in the services provided and catchment population. While the EMS manages acute cases requiring prompt lifesaving intervention, the ED also treats cases that are not considered life threatening 11.

**Table 4:** Comparison with previous CCARE studies in different settings and a Canadian EMS study

Variable	Current study	Asai et al. 2014 7	Ben‐Shoshan et al. 2013 6	Kane et al. 5
Location	Outaouais, Quebec, Canada	Montreal, Quebec, Canada	Montreal, Quebec, Canada	Toronto, Ontario, Canada
Population	Patients requiring EMS	Adults, tertiary care ED	Children, tertiary care ED	Patients requiring EMS
Method	Prospective	Retrospective cases identified through ICD‐10 codes	Retrospective and prospective cases identified through ICD‐10 codes	Retrospective
Total population	All ambulance calls: 23,486	All ED visits: 37,730	All ED visits: 81,677	All ambulance calls: 210,633
Cases	104	98	168	934
Incidence estimate, % (95%CI)	0.44 (0.36, 0.54)	0.26 (0.21, 0.32)	0.21 (0.18, 0.24)	0.44 (0. 42, 0.47)

**Figure 1:**
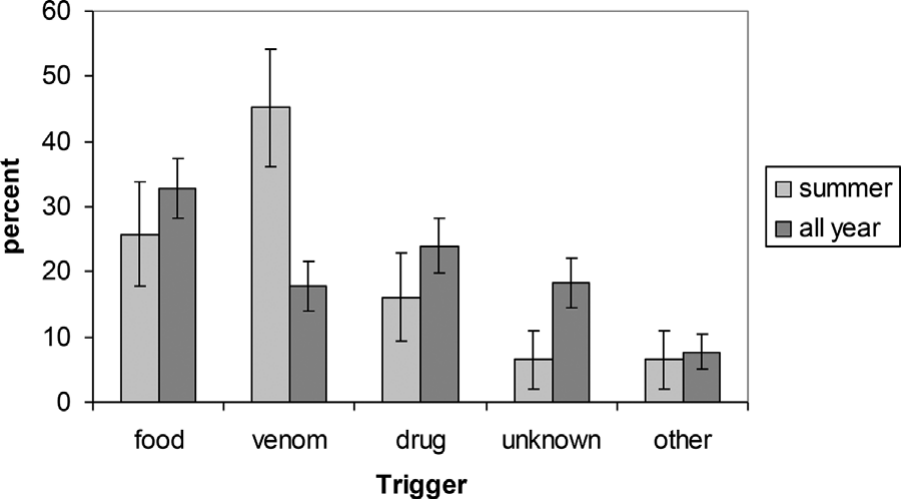
Anaphylaxis triggers during the summer months (June–August) versus all year.

Similar to our studies in pediatric and adult EDs, food was the main trigger 7, 8. However, in our EMS study, food was suspected to trigger 32.7% of reactions, whereas in the pediatric ED, it triggered 86.9% of reactions (difference: −51.2% [95%CI, −62.6%, −39.9%]) and in the adult ED, food triggered 63.7% of reactions (difference: −30.6% [95%CI, −44.7%, −16.5%]). Venom was responsible for 17.3% of reactions in our EMS study versus 3.6% (difference:13.7% [95%CI, 5.2%, 22.3%] in a pediatric and 4.1% [difference:13.2% [95%CI, 4.0%, 22.5%]) in an adult ED. Moreover, when we restricted our analysis to the summer months (May–August), we found that venom was the major culprit for anaphylaxis (45.2% [31.0%, 62.4%] of all reactions) in the EMS setting (Fig. 1). Other studies report higher rates of venom‐induced anaphylaxis during the summer in rural settings 5, 12.

Despite the presence of guidelines stipulating epinephrine use in all cases of anaphylaxis and dedicated training programs for epinephrine utilization by health care professionals 13, a substantial proportion of cases (35.6% [95%CI, 26.6%, 45.6%]) were not treated with epinephrine. Similarly, studies conducted in a variety of countries also found poor adherence to guidelines 14, 15. Specifically, the underuse of epinephrine by paramedics was noted in a recent retrospective study concerning the EMS in Edmonton, Alberta, Canada 5. In the latter, almost 50% did not receive epinephrine (46.3% [37.8%, 55.1%]). Given that 79% of cases in our EMS study were adults, it is possible that the underuse of epinephrine is due to concerns related to side effects of epinephrine administration in older individuals especially with cardiovascular disease. The overall consensus, though, is that the benefits of administering epinephrine, regardless of age, usually outweigh the risks 16. In a recent systemic review, it was concluded that there is no absolute contraindication to epinephrine injection in anaphylaxis 17. Equally noteworthy is the fact that participants treated by the EMS in our study were relatively young (median age: 46.8 years) and less than 5% had a history of cardiovascular disease or use of beta blockers (Table 1). Prompt use of epinephrine is crucial especially in the particular study setting given that the average response time in this area is at least 18 minutes 18.

Another important observation from our study is that only 50% of patients who had an auto‐injector used it at the time of reaction. These findings are also comparable to our pediatric study that found that the majority of parents (56%) expressed fear regarding the use of the EAI mainly because they were concerned they would hurt the child or use the auto‐injector incorrectly 19.

The association of venom‐induced anaphylaxis and use of epinephrine is a new finding. Although the risk of anaphylaxis fatality is low (less than 1 per million in the general population with an average case fatality rate of 0.3%) 20, it is higher in cases of venom‐induced anaphylaxis compared to other triggers 21, 22. Hence, it is possible that paramedics treating venom‐induced anaphylaxis are more likely to use epinephrine 21, 22.

Limitations of this study include our ability to collect data only from paramedics. We did not collect information regarding management after arrival at the ED. It is possible that some of the reactions required further treatment in the ED and that there were bi‐phasic reactions that we could not assess. In addition, we were not able to confirm the suspected culprit as we did not have access to allergy testing that may have been performed. However, in our study at the Montreal Children's Hospital using the identical questionnaire, we found that in about 90% of the cases, the reported culprit was confirmed by either skin prick testing, measurement of allergen‐specific IgE, or allergen challenge 7. Our sample size is relatively small and hence more conclusive associations could not be determined. Further, our results could not be generalized to the EMS setting in other countries given the difference in health care system, ED accessibility, and ambulance response time 23. Finally, this study focuses on a more rural region of Quebec, Canada, and may not be representative of the entire Quebec or Canadian adult population.

In conclusion, this is the first prospective study to assess anaphylaxis in the EMS setting. Our study reveals high rate of anaphylaxis triggered mainly by food and under use of epinephrine. Training and educational programs targeting paramedics and patients with known food or venom allergy are required to promote prompt use of epinephrine in anaphylaxis.

## Conflict of Interest

Dr. Ben‐Shoshan is the recipient of the Emerging Clinician Scientist award and the Fonds de la recherche en santé du Québec (FRSQ) junior 1 award. Dr. Clarke is the recipient of the Arthritis Society Chair in Rheumatic Diseases at the University of Calgary.
